# Validation of the predictive accuracy of “clinical + morphology nomogram” for the rebleeding risk of ruptured intracranial aneurysms after admission

**DOI:** 10.1186/s41016-022-00274-4

**Published:** 2022-03-01

**Authors:** Jianfei Sui, Nuochuan Wang, Pengjun Jiang, Jun Wu, Qingzhen Wang, Qiaolin Yuan, Hongwei He

**Affiliations:** 1 Department of Radiology, Beijing Tiantan Hospital, Capital Medical University, No. 119 South 4th Ring West Road, Fengtai District, Beijing, 100070 China; 2 Department of Transfusion, Beijing Tiantan Hospital, Capital Medical University, No. 119 South 4th Ring West Road, Fengtai District, Beijing, 100070 China; 3 Department of Neurosurgery, Beijing Tiantan Hospital, Capital Medical University, No. 119 South 4th Ring West Road, Fengtai District, Beijing, 100070 China; 4 China National Clinical Research Center for Neurological Diseases, Beijing, People’s Republic of China; 5 Department of Neurointervention, Beijing Tiantan Hospital, Capital Medical University, No. 119 South 4th Ring West Road, Fengtai District, Beijing, 100070 China

**Keywords:** Intracranial aneurysms, Rupture, Rebleeding, Morphology, Predicting model

## Abstract

**Background:**

Rebleeding can cause a catastrophic outcome after aneurysmal subarachnoid hemorrhage. A clinical + morphology nomogram was promoted in our previous study to assist in discriminating the rupture intracranial aneurysms (RIAs) with a high risk of rebleeding. The aim of this study was to validate the predictive accuracy of this nomogram model.

**Method:**

The patients with RIAs in two medical centers from December 2020 to September 2021 were retrospectively reviewed, whose clinical and morphological parameters were collected. The Cox regression model was employed to identify the risk factors related to rebleeding after their admission. The predicting accuracy of clinical + morphological nomogram, ELAPSS score and PHASES score was compared based on the area under the curves (AUCs).

**Results:**

One hundred thirty-eight patients with RIAs were finally included in this study, 20 of whom suffering from rebleeding after admission. Hypertension (hazard ratio (HR), 2.54; a confidence interval of 95% (CI), 1.01–6.40; *P* = 0.047), bifurcation (HR, 3.88; 95% CI, 1.29–11.66; *P* = 0.016), and AR (HR, 2.68; 95% CI, 1.63–4.41; *P* < 0.001) were demonstrated through Cox regression analysis as the independent risk factors for rebleeding after admission. The clinical + morphological nomogram had the highest predicting accuracy (AUC, 0.939, *P* < 0.01), followed by the bifurcation (AUC, 0.735, *P* = 0.001), AR (AUC, 0.666, *P* = 0.018), and ELAPSS score (AUC, 0.682, *P* = 0.009). Hypertension (AUC, 0.693, *P* = 0.080) or PHASES score (AUC, 0.577, *P* = 0.244) could not be used to predict the risk of rebleeding after admission. The calibration curve for the probability of rebleeding showed a good agreement between the prediction through clinical + morphological nomogram and actual observation.

**Conclusion:**

Hypertension, bifurcation site, and AR were independent risk factors related to the rebleeding of RIAs after admission. The clinical + morphological nomogram could help doctors to identify the high-risk RIAs with a high predictive accuracy.

**Supplementary Information:**

The online version contains supplementary material available at 10.1186/s41016-022-00274-4.

## Background

Rebleeding is a catastrophic event with a high mortality after aneurysmal subarachnoid hemorrhage [1–3]. Patients can be protected from poor outcomes though appropriate surgical intervention [4–8], for the limitation of medical sources, a notable part of patients cannot receive treatment as soon as they are sent to hospital. Considering that rebleeding occurs most within 6 h after the initial hemorrhage [2, 3, 9], it is meaningful to establish a model, through which the ruptured intracranial aneurysms (RIAs) with a high risk of rebleeding could be quickly identified.

There are two main aspects related to the stability of intracranial aneurysms, known as the pathological characteristics of aneurysm wall [10] and the hemodynamic characteristics of aneurysms [11–13]. Considering the strong correlation between pathological characteristics of aneurysm wall and morphological features [14], we established a nomogram model based on clinical and morphological factors (hypertension, bifurcation, irregular shape, and aspect ratio) to identify the RIAs with a high risk of rebleeding after admission [15]. However, the predictive accuracy was not validated by other independent cohorts.

In this study, we retrospectively reviewed the clinical characteristics of patients with RIA from two medical centers and the morphology of RIAs. The aim of this study was to validate the predictive accuracy of “clinical + morphological nomogram.”

## Methods

### Study group and screening criteria

**Fig. 1:**
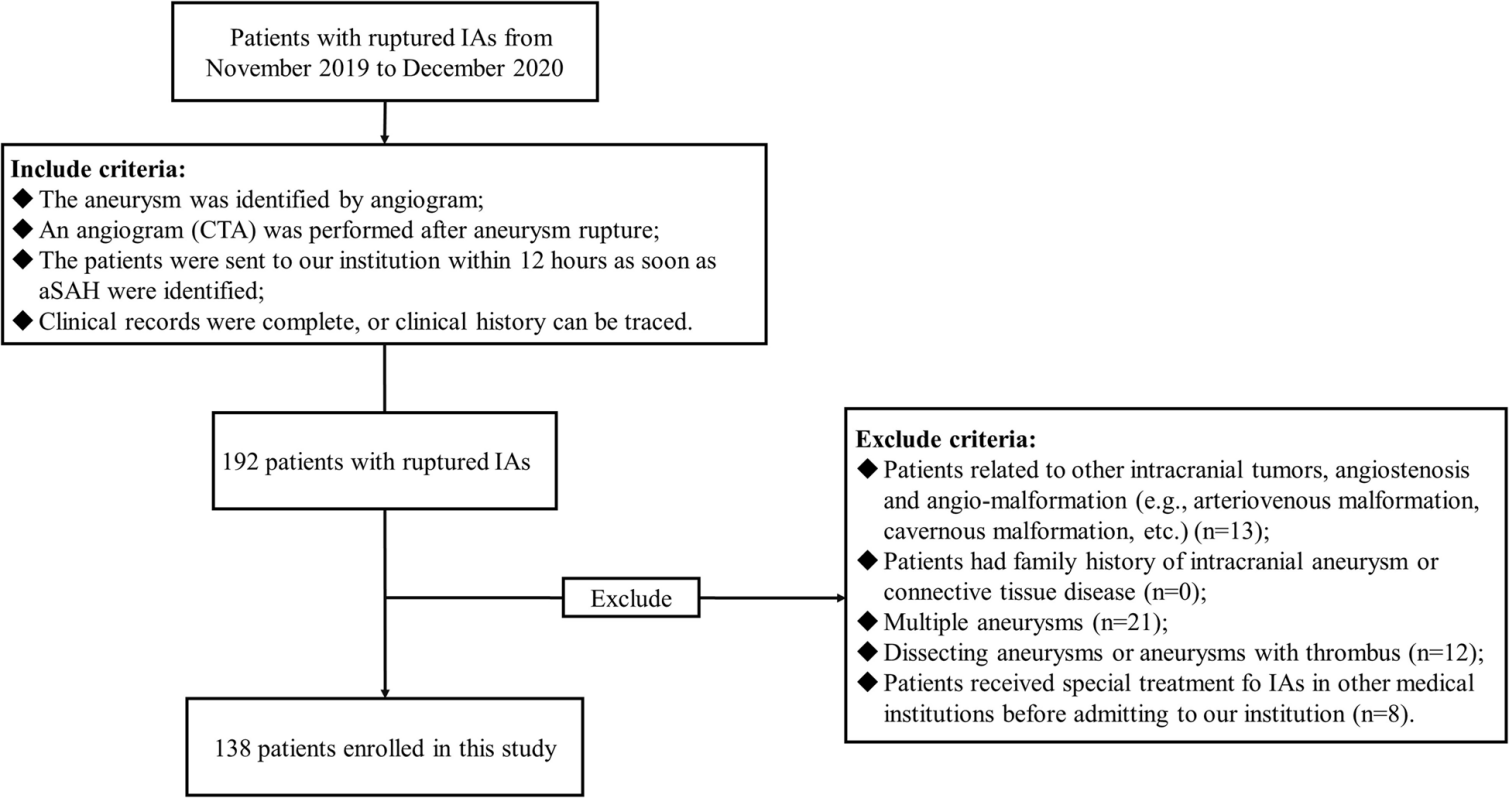
The flow chart. This study enrolled 138 appropriate patients with RIAs from 192 patients. RIA, ruptured intracranial aneurysm

We retrospectively reviewed the patients with RIAs in Beijing Tiantan Hospital and Beijing and Peking University International Hospital from December 2020 to September 2021. As is presented in Fig. 1, patients were selected according to the following standards: (1) a CTA (computational tomography angiogram) was performed after initial hemorrhage; (2) the patients were sent to our institution within 12 h as soon as the occurrence of symptoms, e.g., when an acute headache or a sudden coma occurred; (3) clinical records were complete, or clinical history could be traced. We further excluded the patients who (1) had other intracranial tumors, angiostenosis or angio-malformation, e.g., meningioma or arteriovenous malformation; (2) had a family history of intracranial aneurysm; (3) had multiple intracranial aneurysms, because it would make it difficult to identify the source of bleeding or rebleeding; (4) had dissection, thrombus or traumatic aneurysms; (5) received special treatment for RIA in other medical institutions before admitted to our institution.

### Perioperative management

Once patients were admitted to the medical institutions, they would receive standard medical care, including acute pressure lowering and intensive medical monitoring, followed by the guideline and recommendation [16, 17]. The target of blood pressure was to lower their systolic pressure to 120–140 mmHg.

After initial hemorrhage, patients with Hunt-Hess I–II would receive a surgical intervention within 72 h, who could not benefit from immediate surgical treatment; therefore, we would just give these patients conservative treatment until the Hunt-Hess grade “degraded”; however, once patients’ condition progressively deteriorated, an emergency surgical intervention would be performed. In addition, for patients with Hunt-Hess V, surgical treatment would not be recommended.

### Data collection

Clinical information was collected from electronical medical records regarding to age, gender, comorbidities (including hypertension, dyslipidemia, diabetes mellitus, coronary heart disease, and ischemic stroke), aneurysmal subarachnoid hemorrhage history, Hunt-Hess grade at admission, time from admission to rebleeding (time from admission to neurological symptoms) or surgery as well as blood pressure at admission and before rebleeding/surgery. The time interval from admission to rebleeding or surgery was recorded. Modified Fisher scale (mFS) and IA site were collected based on radiological data.

The digital imaging and communications on medicine data of CTA performed after admission were collected and converted into reordered slices (about 0.5 mm per slice). The DICOM data was introduced into Mimics 17.0 (Mimics Research 17.0, Materialize, Belgium) and reconstructed for further studies.

The measurement of morphological parameters was performed by the same neurosurgeons (PJ.J. and J.W.) based on the vascular model. The discrepancies were solved by consulting a senior neuro-interventionist (HW.H.). Aneurysm size (S), diameter of dome (D), perpendicular height (H), diameter of parent artery, vessel angle (VA), aneurysm inclination angle (AA), volume, and surface area were measured here [18]. The parameters mentioned were measured twice by two investigators, the average of whose measurements was taken for further analyses. Aspect ratio (AR), size ratio (SR), undulation index (UI), and nonsphericity index (NSI) were calculated [19]. An irregular shape was identified according to our previous study [15].

The nomogram points were calculated based on hypertension, AR and bifurcation site, which were then transferred into risk probability or rebleeding according to our previous study [15]. The risk probability of rebleeding was used for further analyses.

### Identification of rebleeding after admission

In this study, rebleeding was diagnosed based on two aspects: (1) the patients had a sudden disorder of consciousness, a gradually worsening neurological state or convulsion after admission, and (2) the magnitude of subarachnoid, intracerebral or intraventricular blood significantly increased in immediate CTs compared with that at admission, and the magnitude of bleeding did not increase or kept stable at/before admission. Rebleeding was confirmed by experienced neurosurgeons (S.W.) according to the bleeding presentation on medical record and CT after admission.

The patients suffering from rebleeding were categorized as the rebleeding group, otherwise the stable group.

### Statistical analysis and model establishment

Categorical variables were presented as numbers (*n*) and percentage (%). Continuous variables with a normal distribution were presented as means and standard deviation, and medians (m) as well as inter-quartile range (IQR) if possible. We compared the differences between continuous variables of the two groups based on Student’s *t* tests or Wilcoxon rank sum tests as well as the differences in categorical variables based on chi-square tests or Fisher’s exact tests. The clinical + morphological nomogram was given in our previous study [15]; the risk of rebleeding after admission was calculated, meanwhile PHASES score and ELAPSS score were calculated according to previous protocols [20, 21]. The parameters with significance in univariable analysis were input into Cox regression model to identify the independent risk factors, whose result was presented as hazard ratio (HR) and a confidence interval of 95% (CI). A two-tailed *P* < 0.05 was considered statistically significant, the SPSS 24.0 (SPSS, Chicago, IL) was adopted for statistical analyses, and a two-sided *P* < 0.05 was considered as statistical significance.

A calibration curve was derived to assess the calibration of the actual rebleeding percentage through the model. The nomogram was subjected to bootstrapping validation (1000 bootstrap resamples). The predictive accuracy of the model while predicting rebleeding was measured by the area under the curve (AUCs) through receiver operating characteristic curve (ROC) analyses. The models with AUC > 0.7 were considered as useful models for clinical work.

## Results

### The differences between rebleeding group and stable group

**Fig. 2:**
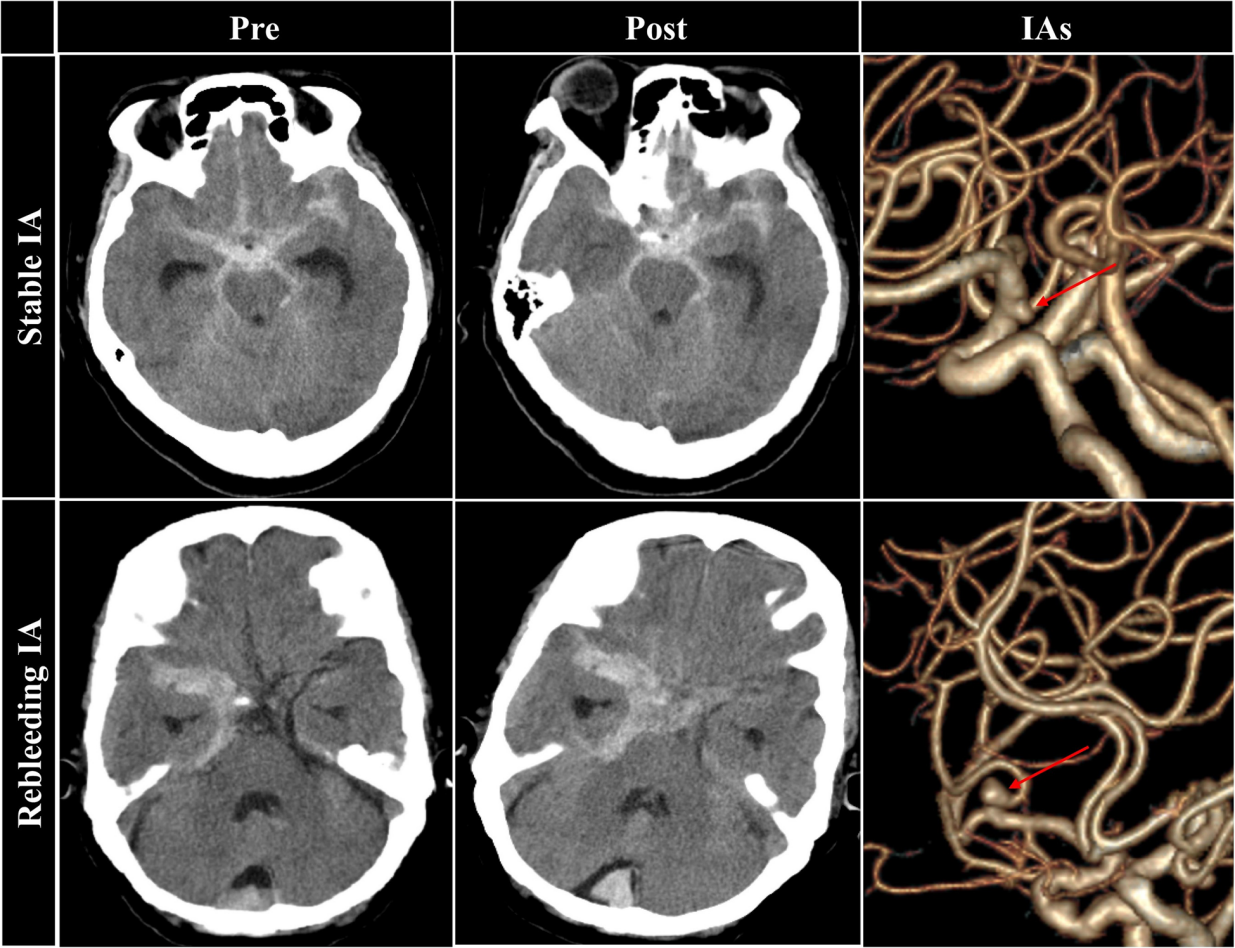
The representative cases. Two representative cases from the rebleeding group and stable group were presented here. The red arrows indicated intracranial aneurysms. IA, intracranial aneurysm

This study finally included 138 patients with RIAs, 20 (14.5%) of whom suffered from rebleeding after admission (Fig. 2). The information of patients and RIAs was summarized in Table 1. For all patients, within those whose age ranged from 21 to 77 years, 57 (41.3%) were male and 47 (34.1%) had a hypertension history. Sixty-five (47.1%) RIAs sited in the internal carotid artery, 49 were (25.5%) in the middle cerebral artery, and 24 (17.4%) sited in other sites.

The significance was found in gender (*P* = 0.002), hypertension (*P* = 0.033), irregular shape (*P* = 0.009), bifurcation (*P* < 0.001), aneurysm size (*P* = 0.005), AA (*P* = 0.014), AR (*P* < 0.001), SR (*P* = 0.018), bottleneck factor (*P* = 0.014), and height-to-width ratio (*P* = 0.002). There was no significance in dyslipidemia, diabetes mellitus, mFS scale, Hunt-Hess grade, locations, dome diameter, height, VA, volume, surface area, or NSI (all *P* > 0.05).

The ELAPSS score was significantly different between rebleeding group and stable group (14 *vs.* 9, *P* = 0.009), whereas the PHASES score failed to be significant (2 *vs.*2, *P* = 0.237).

### Risk factors related to the rebleeding after admission

The result of Cox regression analysis was summarized in Table 2. We performed univariate Cox analyses based on the parameters with significance in univariate analysis. The result showed that hypertension, bifurcation, AR, irregular shape, aneurysm size, AR, SR, bottleneck factor, and height-to-width ratio were risk factors for rebleeding after admission, which were then input into a multivariate model through the backward method. The parameters, including hypertension (HR, 2.54; 95% CI, 1.01–6.40; *P* = 0.047), bifurcation (HR, 3.88; 95% CI, 1.29–11.66; *P* = 0.016), and AR (HR, 2.68; 95% CI, 1.63–4.41; *P* < 0.001), were demonstrated as independent risk factors related to rebleeding after admission.

### The predictive accuracy for the rebleeding after admission

**Fig. 3:**
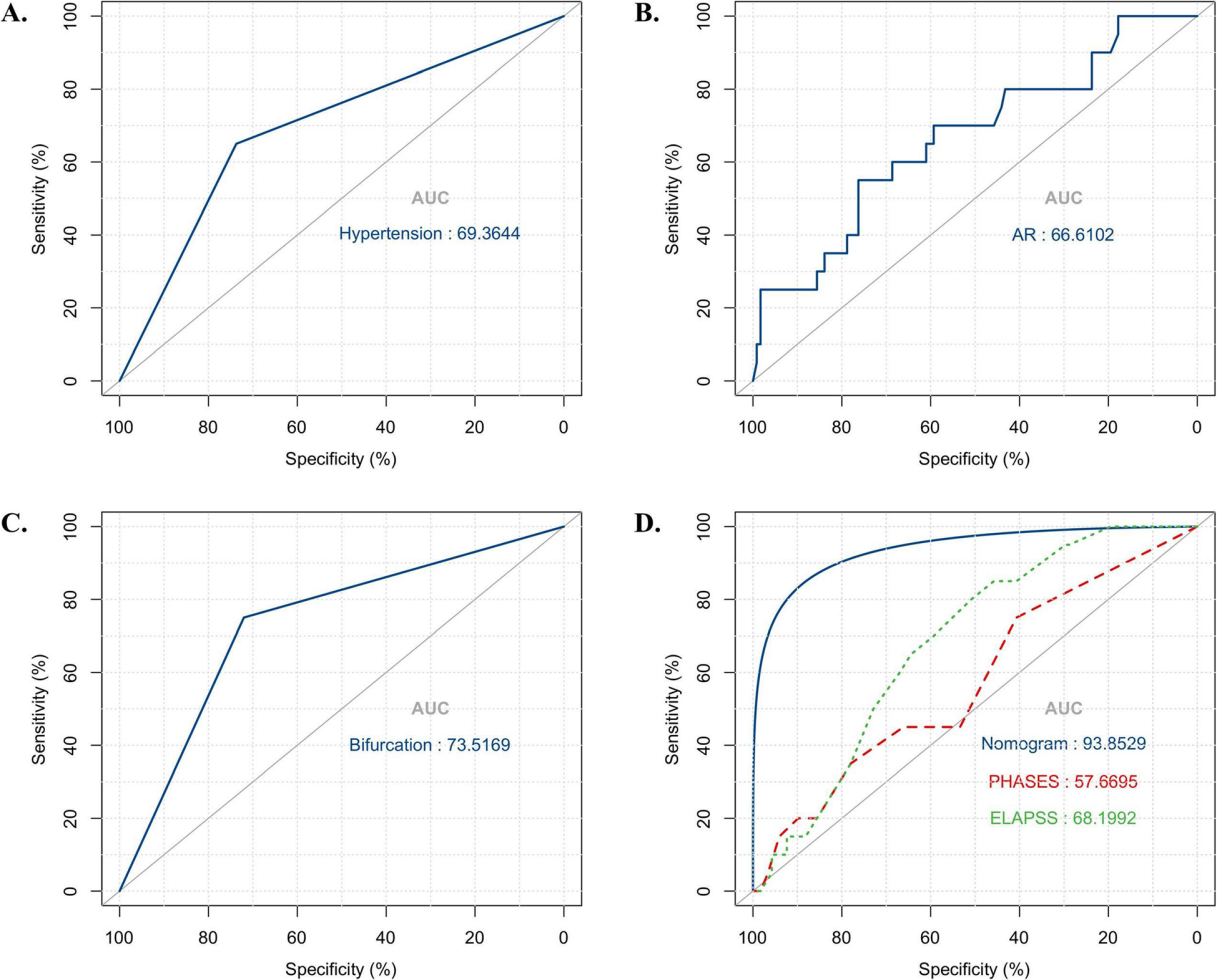
The predictive accuracy. **A**–**C** The predictive accuracy of hypertension, AR and bifurcation for rebleeding. **D** The clinical + morphology nomogram had highest predictive accuracy (AUC, 0.94), followed by ELAPSS score (AUC, 0.68), but PHASES score failed to predict the risk of rebleeding. AUC, area under the curve

**Fig. 4:**
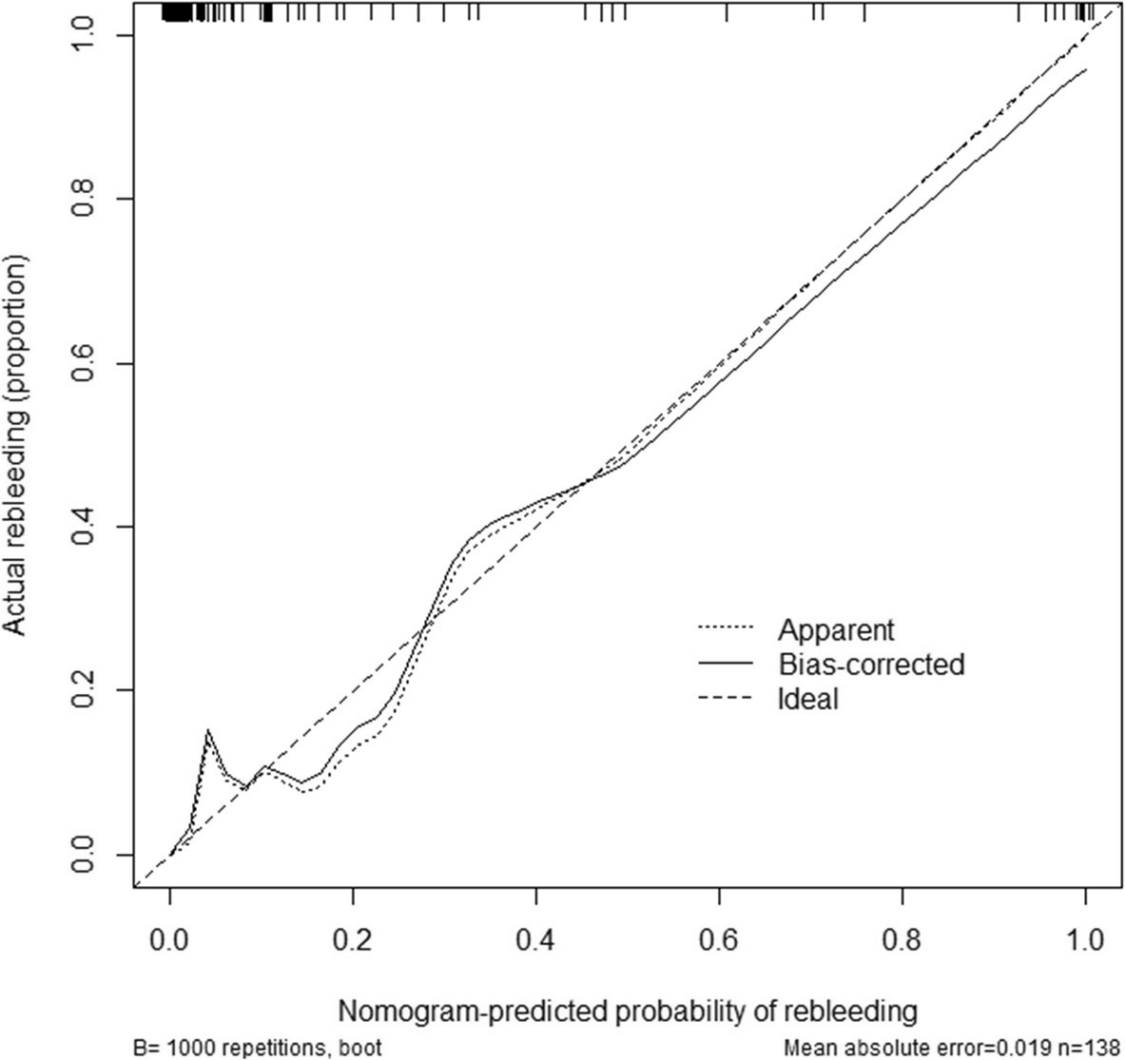
The calibration analysis. The calibration showed a substantial agreement between the prediction by nomogram and the actual observation, in the risk of rebleeding after admission

We further compared the predictive accuracy of independent risk factors, clinical + morphological nomogram, ELAPSS, and PHASES of rebleeding after admission. According to the result of ROC analyses (Fig. 3A–D and Table 3), the nomogram had the highest predictive accuracy (AUC, 0.939, *P* < 0.001), followed by bifurcation (AUC, 0.735, *P* = 0.001) and AR (AUC, 0.666, *P* = 0.018). However, hypertension (AUC, 0.693, *P* = 0.080) and PHASES score (AUC, 0.577, *P* = 0.244) could not be used to predict the risk of rebleeding after admission. For the risk of rebleeding after admission, the calibration plot showed a substantial agreement between the prediction by nomogram and the actual observation (Fig. 4). To avoid the effect of Hunt-Hess grade and time from admission to rebleeding or surgery, we performed a subgroup analysis, whose result showed that rebleeding RIAs had a higher risk probability in both I–II-grade and III–IV-grade patients (Supplementary Figure 1) on each day (Supplementary Figure 2).

## Discussion

Rebleeding is a catastrophic event after aneurysmal subarachnoid hemorrhage. How to identify RIAs with a high risk of rebleeding is helpful to make treatment strategies. This study demonstrated hypertension, bifurcation and AR as independent risk factors related to rebleeding after admission. We validated the predictive accuracy of the clinical + morphological nomogram for the risk of rebleeding.

Hypertension can aggravate the unstable hemodynamic condition of intracranial aneurysms [20–22]. In the current study, we found that hypertension could increase the risk of rebleeding after admission, which was consistent with our previous study [15]. The potential mechanism is that a systematic hypertension can damage the vascular structure and make the vessels fragile [22–24]. Such biological effect makes the vessels prone to rupture once an accidental stress occurs.

It was also confirmed in this study that the aneurysms sited in bifurcation or with a large AR had a high risk of rebleeding after admission, and that the aneurysms sited in bifurcation had a higher possibility to suffer from the impact of blood flow [13, 25]. The dynamic change from direct-impact area to surrounding area could cause physical injuries to the aneurysm wall [13]. The aneurysms with a large AR usually are in an unstable hemodynamic condition with severe damage in aneurysm wall [26]. In our preliminary studies, we also found that AR was also a predictor for rebleeding of RIAs [14]. Based on these facts, bifurcation site and AR could serve as the parameters to identify the RIAs with a high risk of rebleeding after admission.

In previous study, we established a nomogram based on clinical and morphological characteristics of RIAs, including hypertension, bifurcation, irregular shape and aspect ratio, which were also confirmed as the independent risk factors for rebleeding in this study. It was revealed in our further analyses that the clinical + morphological nomogram reached a high predictive accuracy, which outperformed independent risk factors, ELAPSS and PHASES model. This model could help neurosurgeons and neuro-interventionists quickly identify the rebleeding risk of RIAs based on CTA, which was also easy to follow and understand. In China, because of a large patient population with limitations of medical resources [27], it is meaningful to give priority to RIAs with a high risk of rebleeding. Therefore, the clinical + morphological nomogram could assist doctors to make treatment strategies.

There were two limitations in the current study. First of all, because of the change in morphology after indiscoverable rebleeding [28] and the effect of hemorrhage on the quality of radiological images, our conclusion may be limited. Secondly, not so many potential factors were considered this study, which might be also related to the risk of rebleeding. Though there are some limitations, this study could also provide the evidence that clinical + morphological nomogram could help doctors to identify the RIAs with a high risk of rebleeding.

## Conclusion

Hypertension, bifurcation site, and AR were independent risk factors related to rebleeding of RIAs after admission. The clinical + morphological nomogram could help doctors to identify the high-risk RIAs with a high predictive accuracy.

## Supplementary Information

**Additional file 1:** Supplementary Figure 1. Subgroup analysis based on Hunt-Hess grade. We performed subgroup analysis based on Hunt-Hess grade, and found that the rebleeding RIAs had higher risk probability in both I-II grade and III-IV grade patients.

**Additional file 2:** Supplementary Figure 2. Subgroup analysis based on operation time. We performed subgroup analysis based on operation time. 96 patients received surgery at the first day, 37 received surgery at the second day, and 5 received surgery at the third day. Comparing with the stable RIAs, the rebleeding RIAs had higher risk probability in each day.

## Data Availability

Data sharing not applicable to this article as no datasets were generated or analyzed during the current study.

## Abbreviations

RIA: Ruptured intracranial aneurysm
CT: Computational tomography
CTA: Computational tomography angiography
Mfs: Modified Fisher scale
S: Aneurysm size
D: Diameter of dome
H: Perpendicular height
VA: Vessel angle
AA: Aneurysm inclination angle
AR: Aspect ratio
SR: Size ratio
UI: Undulation index
NSI: Nonsphericity index
