# Supplementary material for: Validation of the predictive accuracy of “clinical + morphology nomogram” for the rebleeding risk of ruptured intracranial aneurysms after admission
Source: Chin Neurosurg J. 2022 Mar 1;8:5. doi: 10.1186/s41016-022-00274-4 (PMC8886787; doi:10.1186/s41016-022-00274-4)
Supplement: Supplementary file 1 — Additional file 1: Supplementary Figure 1. Subgroup analysis based on Hunt-Hess grade. We performed subgroup analysis based on Hunt-Hess grade, and found that the rebleeding RIAs had higher risk probability in both I-II grade and III-IV grade patients. [file 41016_2022_274_MOESM1_ESM.pdf]

Supplementary to *Validation of the predictive accuracy of "Clinical + Morphology nomogram" for the rebleeding risk of ruptured intracranial aneurysms after admission*

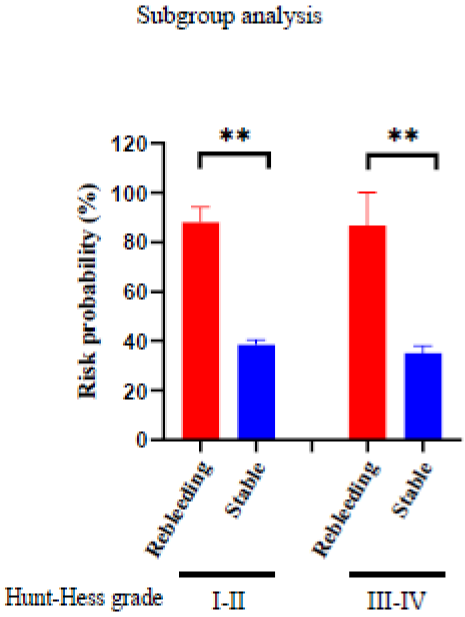

Supplementary Figure 1. Subgroup analysis based on Hunt-Hess grade. We performed subgroup analysis based on Hunt-Hess grade, and found that the rebleeding RIAs had higher risk probability in both I-II grade and III-IV grade patients.
