# Supplementary material for: Validation of the predictive accuracy of “clinical + morphology nomogram” for the rebleeding risk of ruptured intracranial aneurysms after admission
Source: Chin Neurosurg J. 2022 Mar 1;8:5. doi: 10.1186/s41016-022-00274-4 (PMC8886787; doi:10.1186/s41016-022-00274-4)
Supplement: Supplementary file 2 — Additional file 2: Supplementary Figure 2. Subgroup analysis based on operation time. We performed subgroup analysis based on operation time. 96 patients received surgery at the first day, 37 received surgery at the second day, and 5 received surgery at the third day. Comparing with the stable RIAs, the rebleeding RIAs had higher risk probability in each day. [file 41016_2022_274_MOESM2_ESM.pdf]

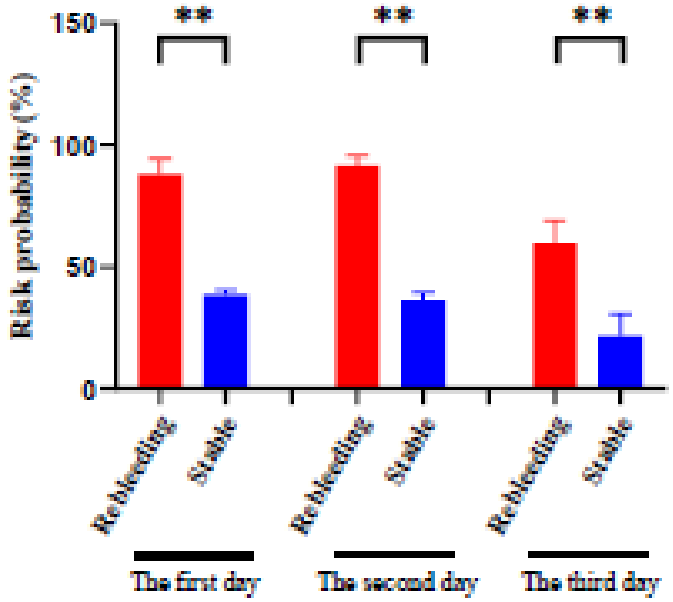

Supplementary Figure 2. Subgroup analysis based on operation time. We performed subgroup analysis based on operation time. 96 patients received surgery at the first day, 37 received surgery at the second day, and 5 received surgery at the third day. Comparing with the stable RIAs, the rebleeding RIAs had higher risk probability in each day.
